# Overexpression of PGC-1**α** Increases Fatty Acid Oxidative Capacity of Human Skeletal Muscle Cells

**DOI:** 10.1155/2012/714074

**Published:** 2011-09-04

**Authors:** Nataša Nikolić, Magdalena Rhedin, Arild C. Rustan, Len Storlien, G. Hege Thoresen, Maria Strömstedt

**Affiliations:** ^1^Department of Pharmaceutical Biosciences, School of Pharmacy, University of Oslo, P.O. Box 1068 Blindern, N-0316 Oslo, Norway; ^2^AstraZeneca Reseach and Development, SE-43185 Mölndal, Sweden; ^3^Boden Institute of Obesity, Nutrition and Exercise, University of Sydney, Sydney, NSW 2006, Australia

## Abstract

We investigated the effects of PGC-1**α** (peroxisome proliferator-activated receptor **γ** coactivator-1**α**) overexpression on the oxidative capacity of human skeletal muscle cells *ex vivo*. PGC-1**α** overexpression increased the oxidation rate of palmitic acid and mRNA expression of genes regulating lipid metabolism, mitochondrial biogenesis, and function in human myotubes. Basal and insulin-stimulated deoxyglucose uptake were decreased, possibly due to upregulation of PDK4 mRNA. Expression of fast fiber-type gene marker (MHCIIa) was decreased. Compared to skeletal muscle *in vivo*, PGC-1**α** overexpression increased expression of several genes, which were downregulated during the process of cell isolation and culturing. In conclusion, PGC-1**α** overexpression increased oxidative capacity of cultured myotubes by improving lipid metabolism, increasing expression of genes involved in regulation of mitochondrial function and biogenesis, and decreasing expression of MHCIIa. These results suggest that therapies aimed at increasing PGC-1**α** expression may have utility in treatment of obesity and obesity-related diseases.

## 1. Introduction

The genesis of obesity is multifactorial. However, there is evidence that reduced energy expenditure and in particular reduced capacity to utilise fat for metabolic fuel are important factors, particularly in the weight reduced state [1] PGC-1*α* (peroxisome proliferator-activated receptor *γ* coactivator-1*α*) is a transcriptional coactivator initially isolated from brown adipose tissue [2], but now known to be abundant in many metabolically active tissues, such as skeletal muscle, liver, heart, and brain, where PGC-1*α* plays a major role in transduction of nutritional and physiological stimuli to transcriptional metabolic and contractile responses [2, 3]. 

Among many transcription factors coactivated by PGC-1*α* are nuclear respiratory factors (NRF1/2) [4], myocyte enhancer factor-2 (MEF2) [4], and several members of nuclear hormone receptors, including peroxisome proliferator-activated receptor (PPAR) subtypes—a family of lipid activated nuclear hormone receptors that play a key role in mediating adaptive regulation of muscle fatty acid oxidation [5]. The most common function of PGC-1*α* across tissues is regulation of mitochondrial physiology, but in addition, this family of coactivators controls separate, tissue-specific biological programs. In liver, expression of PGC-1*α* is strongly induced by fasting and stimulates hepatic gluconeogenesis and ketogenesis [6, 7]; in heart, it is a powerful stimulant of mitochondrial gene expression and biogenesis [8], while PGC-1*α* deficiency in brain has been shown to lead to behavioral abnormalities associated with axonal degeneration [9]. 

In skeletal muscle, PGC-1*α* is powerfully induced in conditions of increased physical activity [10–15], when ATP demand is high and induction of mitochondrial oxidative function becomes essential in order to adapt and maintain whole body energy balance. Enhancement of mitochondrial function and biogenesis occur through PGC-1*α* coactivation of nuclear respiratory factor (NRF) [16], and regulation of genes involved in oxidative phosphorylation is mediated through interactions with estrogen-related receptor *α* (ERR*α*) [17]. In addition, adaptation to increased contractile activity involves conversion from type II (fast twitch) to type I (slow twitch) fibers [18], a process also shown to be driven by PGC-1*α* in transgenic animals [16], while muscle-specific knock out of PGC-1*α* in animals has been shown to lead to conversion from type I to type II muscle fibers, exercise intolerance, decrease in mitochondrial proteins and myopathy [19]. Also in primary skeletal muscle cells from rats, overexpression of PGC-1*α* has been shown to confer a switch toward a more slow myofiber phenotype [20]. Further, overexpression of PGC-1*α* improved lipid utilization, insulin signaling and glucose transport *in vivo* animal studies [21, 22], while whole body overexpression of PGC-1*α* appears to have opposite effects on hepatic and muscle insulin sensitivity [23]. In cell cultures of rodent myotubes (C2C12 and L6), overexpression of PGC-1*α* has been shown to increase the level of the insulin-regulated glucose transporter 4 (GLUT4) mRNA and glucose uptake [24]. 

Peroxisome proliferator-activated receptor *δ* (PPAR*δ*) is coactivated by PGC-1*αin vivo *and *in vitro*, and has been linked to increased fatty acid oxidation [25]. In addition, PPAR*δ*, along with PGC-1*α*, has been identified as one of key players mediating effects of exercise at the cellular level [16, 26, 27] and suggested as a new target for the treatment of metabolic syndrome [28].

Cultured myotubes are a useful tool in investigating metabolic processes at cellular level, but limited in their usefulness in that they are characterized by glycolytic properties [29, 30]. PGC-1*α* has been referred to as “master regulator” of the coordination of mitochondrial biogenesis, mainly because increase in PGC-1*α* has been shown to activate transcription factors that switch muscle cells towards oxidative metabolism [31–33]. However, significant reduction of PGC-1*α* levels in muscle cell cultures compared to *in vivo *muscle extracts has been reported in chicken [34], and the role of PGC-1*α* in metabolic processes in skeletal muscle *in vitro* has previously been highlighted using adenoviral overexpression in cell cultures [24], but data describing metabolic effects of PGC-1*α* overexpression in primary human skeletal muscle cells are limited [35]. 

 The aim of the present work was to study whether overexpression of PGC-1*α* in cultured human skeletal muscle cells from healthy individuals would increase the oxidative capacity of the cells, promote fiber type conversion, and increase expression of genes involved in mitochondrial biogenesis and function. Moreover, we wanted to compare cultured myotubes to skeletal muscle *in vivo.* Finally, a possible interplay between PGC-1*α* overexpression and pharmacological activation of PPAR*δ* on fatty acid oxidation and expression of key genes in lipid metabolism was studied.

## 2. Materials and Methods

### 2.1. Materials

DMEM-Glutamax, FCS, Ultroser G, penicillin-streptomycin-amphotericin B, and trypsin-EDTA were obtained from Life Technology (Paisley, UK). Skeletal Muscle Growth Medium Bullet Kit was obtained from Clonetics (BioWittaker, Verviers, Belgium). [^3^H]palmitic acid (2.0 GBq/mmol) and 2-deoxy-D-[^3^H]glucose (222 GBq/mmol) were purchased from Dupont NEN Life Science Products (Boston, MA, USA). Palmitic acid, BSA (essentially fatty acid-free), Cytochalasin B, and extracellular matrix gel were purchased from Sigma Chemicals (St Louis, MO, USA). Insulin Actrapid was from Novo Nordisk (Bagsvaerd, Denmark). RNeasy Mini kit and RNase-free DNase were purchased from Qiagen Sciences (Oslo, Norway). Primers were purchased from Invitrogen (Oslo, Norway). High capacity cDNA archive kit, SYBR Green, TaqMan reverse-transcription reagents kit, TaqMan Universal PCR Master Mix and micro fluidic cards were purchased from Applied Biosystems (Warrington, UK). Protein assay kit was purchased from BioRad (Copenhagen, Denmark). All other chemicals used were of standard commercial high purity quality.

### 2.2. Human Skeletal Muscle Biopsies and Cell Cultures

Muscle biopsy samples of the musculus obliquus internus abdominis or vastus lateralis were taken from seven healthy volunteers, and a cell bank of satellite cells was established. The biopsies were obtained with informed consent and approval by the National Committee for Research Ethics (Oslo, Norway). Muscle cell cultures free of fibroblasts were established by the method of Henry et al. [36]. Briefly, muscle tissue was dissected in Ham's F-10 medium at 4°C and dissociated by three successive treatments with 0.05% trypsin/EDTA, and satellite cells were resuspended in skeletal muscle cell growth medium 2% FCS, 50 U/mL penicillin, 50 *μ*g streptomycin, 1.25 *μ*g/mL amphotericin B, and no added insulin. The cells from each donor were grown separately on culture wells or flasks coated with extracellular matrix gel [37]. After 1-2 weeks, at ~80% confluence, growth medium was replaced by DMEM with 2% FCS, 50 U/mL penicillin, 50 *μ*g/mL streptomycin, 1.25 *μ*g/mL amphotericin B, and 25 pM insulin to induce differentiation of myoblasts into multinucleated myotubes. The cells were cultured in a humidified 5% CO_2_ atmosphere at 37°C, and medium was changed every 2-3 days. All myotube cultures were used for analysis on day 7 or 8 after the onset of differentiation.

### 2.3. Transient Retroviral Vector Production

The three retroviral constructs used in this study were pBABE (empty vector), pBABE-hPGC-1*α*, and pBABE-zsGreen (positive control for infection efficacy). 293T/17 cells were seeded 8 h prior to transfection at a density of 4∗10^6^ cells per 100 mm culture dishes in 15 mL of DMEM with 2 mM L-glutamine and 10% foetal calf serum (FCS). Transfection was performed using ProFection Mammalian Transfection System (Promega Biotech, Sweden). DNA mixture was prepared by diluting following amounts of plasmids: 16 *μ*g genome, 16 *μ*g pVPack and 8 *μ*g pME-VSV-G to 438 *μ*L distilled water per 100 mm culture dish. Fifteen min prior to transfection, 65.5 *μ*L CaCl_2_ and 500 *μ*L HEPES were added to DNA mixture, and 1 mL of DNA/CaCl_2_/HEPES was then added to each culture dish. The cells were allowed to incubate at a humidified 5% CO_2_ atmosphere at 37°C for 24 h, when medium was replaced with 10 mL fresh prewarmed DMEM with 10 mM sodium butyrate (per 100 mm culture dish) and incubated at 5% CO_2_ atmosphere and 37°C for an additional 6 h. The medium was then removed, and fresh medium without sodium butyrate was added, and the cells incubated for another 16 h. The medium was collected and the supernatant filtered through 0.45 *μ*m pore filter and used directly to infect cultured myoblasts. The described protocol was applied identically for production of all three retroviral constructs: pBABE, pBABE-hPGC-1*α*, and pBABE-zsGreeen.

### 2.4. Retroviral Infection of Cultured Human Myoblasts

Infection of cultured myoblasts with 100% viral supernatant in the presence of 2 *μ*g/mL polybrene for 24 h gave highest infection efficiency in pilot experiments, and these conditions were chosen for further infections. Cultured myoblasts were seeded at a density of 8000–14000 (depending on growth rate) cells/cm^2^ and infected 1-2 days after seeding with 2 mL (per well in a 6-well plate) 100% retroviral supernatant containing either pBABE (empty vector), PGC-1*α* or pBABE-zsGreen (positive control) in the presence of 2 *μ*g/mL polybrene, and incubated for 24 h. Thereafter, the virus-containing medium was replaced by Clonetics SkGM-BulletKit without insulin. The cells were allowed to reach ~80% confluence, when medium was replaced by DMEM with 2% FCS, 50 U/mL penicillin, 50 *μ*g/mL streptomycin, 1.25 *μ*g/mL amphotericin B and 25 pM insulin to induce the differentiation of myoblasts into multinucleated myotubes. All experiments on myotube cultures were performed on day 7 or 8 after the onset of differentiation. Pretreatments with GW501516 or control (DMSO) were performed 48 h prior to experiments.

### 2.5. Palmitic Acid Uptake and Oxidation

Myotubes grown on six-well plates were rinsed with 3 mL of PBS and exposed to [^3^H]palmitic acid (50 *μ*M, 0.5 *μ*Ci/mL in PBS with 0.5% fatty acid free albumin (BSA) 1 mL/well), in a humidified 5% CO_2_ atmosphere at 37°C. After 30 or 60 min of incubation, myotubes were placed on ice, and the incubation medium was transferred to new vials and assayed for labeled acid-soluble metabolites (ASMs) as previously described [38]. Briefly, incubation medium was precipitated by addition of 150 *μ*L of 20% BSA and 80 *μ*L of 1 M perchloric acid and centrifuged twice (20000 g, 5 min, 4°C). The supernatant was counted for radioactivity by liquid scintillation. No-cell controls were included. Cells were washed twice with PBS and lysed in 0.3 mL M-PER (Mammalian Protein Extraction Reagent, Thermo Scientific). Cell-associated radioactivity was measured by liquid scintillation. PA uptake was calculated as the sum of ASM (corrected for no cell control) in the medium and the cell-associated radioactivity in the cells. Protein content was determined using the BCA assay (Pierce).

### 2.6. Deoxyglucose Transport and Glycogen Synthesis

Myotubes were incubated for 60 min in serum-free DMEM with 5.5 mM glucose, with or without 10 *μ*M cytochalasin B at 37°C. Deoxyglucose uptake was measured for 15 min in the presence of 10 *μ*M unlabeled deoxyglucose and (1 *μ*Ci/mL) 2-deoxy-D-[^3^H]glucose in glucose uptake buffer (140 mM NaCl, 20 mM HEPES, 5 mM KCl, 2.5 mM MgSO_4_, 1 mM CaCl_2_, pH 7.4). After incubation, the cells were washed three times with ice-cold PBS and lysed with 0.3 mL M-PER, and the radioactivity was counted by liquid scintillation. Noncarrier mediated glucose transport was determined in the presence of cytochalasin B (10 *μ*M) and subtracted from all values. For measurement of glycogen synthesis, myotubes were incubated for 2 h (37°C, 5% CO_2_) in serum-free DMEM (±100 nM insulin), before adding DMEM with 2 *μ*Ci/mL D-[U-^14^C]-glucose in the presence or absence of 100 nM insulin for 60 min. After 60 min, the cells were washed three times with ice-cold PBS and lysed with 1 M NaOH. Synthesised glycogen was measured as described [39]. Glycogen synthesis increased linearly within 4 h after insulin stimulation and is presented as nmol*·*mg cell protein^−1^ *·*h^−1^. The protein content of each sample was determined according to Bradford using BSA as the reference protein.

### 2.7. RNA Isolation and Analysis of Gene Expression by Quantitative Real-Time PCR

Human skeletal muscle biopsies and myotubes grown in 6-well plates were lysed in 1 mL Trizole reagent (Invitrogen, Oslo, Norway) according to the supplier's total RNA isolation protocol. The isolated RNA was dissolved in RNase-free water, and concentration was determined by spectrophotometric measurement at 260 nm. All samples were also electrophoresed in 1% agarose gel to assess integrity of ribosomal bands.

Total RNA from above was treated with DNase (DNA-free, Ambion) to remove contaminating genomic DNA before cDNA synthesis. Shortly, 0.1 volume of 10 times DNase buffer, 4–6 units of DNase and water up to 30 *μ*L was added to each sample. Samples were incubated at 37°C for 40–45 min. DNase inactivation reagent was added to each sample and removed by centrifugation after 2 min incubation at room temperature. 

First strand cDNA synthesis was performed with SuperScript III First-Strand Synthesis System for RT-PCR (Invitrogen, Oslo, Norway) according to supplier protocol. 1 *μ*g of DNase treated total RNA was used in each synthesis. Priming of synthesis was accomplished with Oligo(dT)_12-18_. For each synthesis reaction a negative control with no enzyme was set up. Obtained cDNA was used for relative quantification on Applied Biosystems 7700. Quantitative real-time PCR was carried out using either TaqMan Universal PCR Master Mix or SYBR Green PCR Master Mix (both from Applied BioSystems) in 25 *μ*L reactions run in triplicates. Template for the PCR was first strand cDNA from above (amount equivalent of 5–25 ng total RNA). Following forward and reverse primers and in case of TaqMan Universal PCR Master Mix, labelled probes, specific for the different genes, were used at concentrations of 200 nM and 400 nM, respectively: *CD36*: F: GGGAAAGTCACTGCGAC ATGA, R: GAACTGCAATACCTGGCTTTTCTC, Probe: TACAGATGCAGCCTCATTTC CACCTTTTGT; *CPT1b*: F: GCGCTGGAGGTGG CTTT, R: TCGTGTTCTCGCCTGCAAT, Probe: AAACTCCATAGCCATCATCTGCTAC AGGGC; *MCAD*: F: TACTTGTAGAGCACCAAGCAATATCA, R: TGCTCTCTGGTAA CTCATTCTAGCTAGT, Probe: CAACTTTCATTGCCATTTCAGCCAGCATA; *ATGL*: F: CGCACCTGTGCCTTAATCTTC, R: GCTGCAAAGTTCTCAGGAGTAAAG; *HSL*: F: C AGAAGATGTCGGAGCCCATA, R: GGCCAGTGCTGCTTCAGAC; *PPAR*α**: F: CTCT CAGGAAAGGCCAGTAACAA, R: TGGCCACCAGCGTCTTCT, Probe: CACCTTTTG TCATACATGATATGGAGACACTGTGT; *PPAR*γ**: F: GTCACGGAACACGTGCAGC, R: GCAGGAGCGGGTGAAGACT; *PPAR*δ**: F: TGCGGCAACTGGTCACC, R: TCTCGGTT TCGGTCTTCTTGA; *cytochrome C*: F: CTGCCAACAACGGAGCATT, R: CGTGAGCA GGGAGAAGACGTA, Probe: CACCATGCCTAGCTCGCACGATGTAG; *COXIV*: F: CC GCGCTCGTTATCATGTG, R: CACCCACTCTTTGTCAAAGCTTT, Probe: CACTATGT GTACGGCCCCCTCCCG; *SOD2*: F: GCTTGTCCAAATCAGGATCCA, R: GCGTGCTC CCACACATCA; *SOD3*: F: GGCCTCCATTTGTACCGAAA, R: CGGGAGTCTCAGGGC TTATG; *UCP-2*: F: TGAGCTGGTGACCTATGACCTCAT, R: AGTGGCAAGGGAGGTC ATCTGT, Probe: AAGGATGCCCTCCTGAAAGCCAACCT; *UCP-3*: F: CTGCTGGACT ATCACCTGCTCA, R: CCACCACTGTGGCACAGAAG, Probe: ACAACTTCCCCTGCC-ACTTTGTCTCTGC; *ERR*α**: F: GAGAGGAGTATGTTCTACTAAAGGCCTT, R: GCCTC GTGCAGGAGCTTCT, Probe: TCGGCTCATCTTCGATGTGCACAGAGTC, *NRF1*: F: C ATGCGTTGAGCTACTGACAAAC. R: AGTCCAGCAGGGAGAGTTCTGT, *mtTFA*: F: GCTGAAAGATTCCAAGAAGCTAAGG, R: TTCAGAGTCAGACAGATTTTTTTCCAG-TT; *MHCI*: F: CCAGACTGTGTCTGCTCTCTTCAG, R: CAGGACAAGCTCATGCTCC AT, *MHCIIa*: F: AAGGTCGGCAATGAGTATGTCA, R: CAACCATCCACAGGAACAT CTTC.

For all PCR reactions, two types of negative controls were used. As control for possible amplification of remaining genomic DNA, no enzyme controls from cDNA synthesis were set up for all primer-probe sets. The other negative control was with no template included. All signals were normalized to housekeeping gene 36B4. Samples were run on ABI Prism 7700 using the default program: first, two steps with 50°C for 2 min and 95°C for 10 min, followed by 40 cycles of 95°C for 15 sec and 60°C for 1 min. When SYBR Green PCR Master Mix was used, PCR product from one well in each triplicate was loaded on an 2.5% NuSieve Agarose (BioWhittaker Molecular Applications) gel to confirm one band in the correct size. 

### 2.8. Immunoblots

Aliquots of 20 *μ*g cell protein from total cell lysates prepared in Laemmli buffer were electrophoretically separated on NuPage 4–12% (w/v) Bis-Tris gel (Invitrogen), followed by immunoblotting with specific antibodies (Anti OxPhos Complex IV, mouse monoclonal 20E8, Cat. A-21248 from Molecular Probes, Anti Cytochrome C, mouse monoclonal 7H8. 2C12, Cat. 556433 from BD Pharmingen). Immunoreactive bands were visualized with ECL Western blotting detection reagents (Amersham International).

### 2.9. Statistical Analyses

All statistical analyses were performed using GraphPad Prism 4.0 for Windows (GraphPad Software Inc., San Diego, CA). Two-tailed paired *t*-tests were performed to determine the effects of PGC-1*α* overexpression. All values in figures are presented as mean ± SEM. Statistical significance was set at *P* < 0.05. For gene analysis, a fold change ≥2 or ≤0.5 was considered an increase or a decrease in expression level, respectively.

## 3. Results

### 3.1. Retroviral Overexpression of PGC-1*α* in Cultured Human Skeletal Muscle Cells

**Figure 1:**
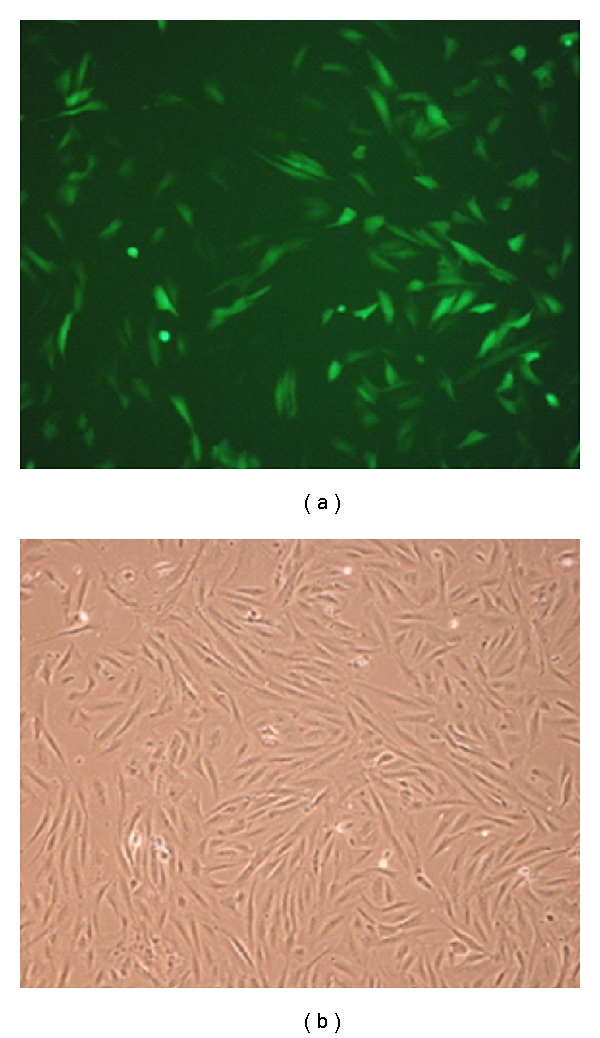
Retrovirus-mediated PGC-1*α* overexpression in human skeletal muscle cells. Cultured myoblasts were infected 24–48 h post seeding with 100% medium containing positive control virus pBABE-zsGreen in the presence of 2 *μ*g/mL polybrene. Cells were incubated in virus-containing medium for 24 h, as described in Section 2. Images were taken 48 h post infection. (a) Fluorescence microscope image. (b) Light microscope image.

**Figure 2:**
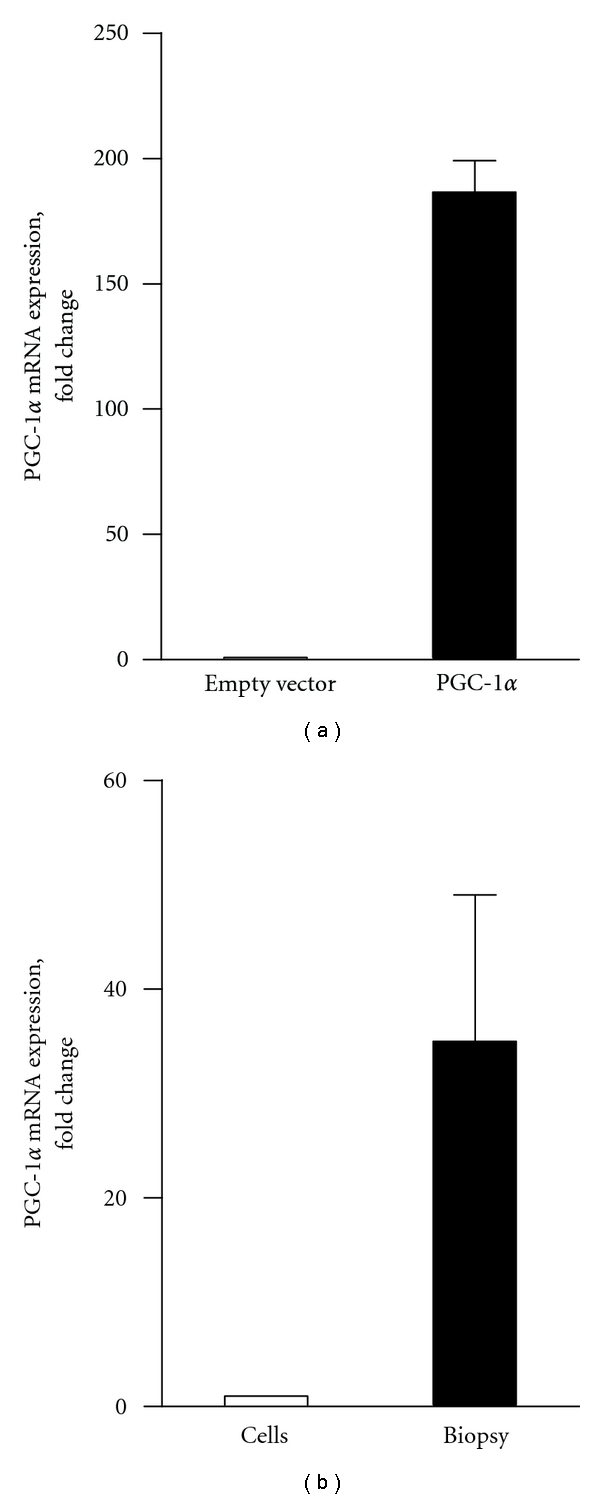
mRNA level of PGC-1*α* in human skeletal muscle cells infected with retrovirus coding for PGC-1*α* compared to control cells infected with empty virus (a), and in muscle biopsies compared to cell cultures (b). mRNA was isolated from muscle biopsies and cultured myotubes, and mRNA expression was assessed by RT-PCR, as described in Section 2. Values are presented as means ± SEM of 3 experiments, each representing one donor, with 3 replicates each, normalized to levels of housekeeping gene 36B4.

To determine whether experimental alterations in PGC-1*α* expression would affect oxidative capacity of cultured human skeletal muscle cells, myoblasts were infected 48 h after seeding. Images of myoblasts taken 48 h after infection with positive control virus pBABE-zsGreen are shown in Figure 1. No morphological changes compared to uninfected control cells were observed in cells infected with either empty vector or PGC-1*α* (data not shown). PGC-1*α* mRNA expression in cells infected with PGC-1*α* increased 150–200-fold compared to cells infected with empty control vector (Figure 2(a)). In skeletal muscle biopsies, PGC-1*α* mRNA expression was about 35-fold higher than in differentiated cultured skeletal muscle cells (Figure 2(b)), thus PGC-1*α* infection restored the reduction in PGC-1*α* mRNA expression observed during cell culturing.

### 3.2. PGC-1*α* Overexpression Increased Palmitic Acid Oxidation

**Figure 3:**
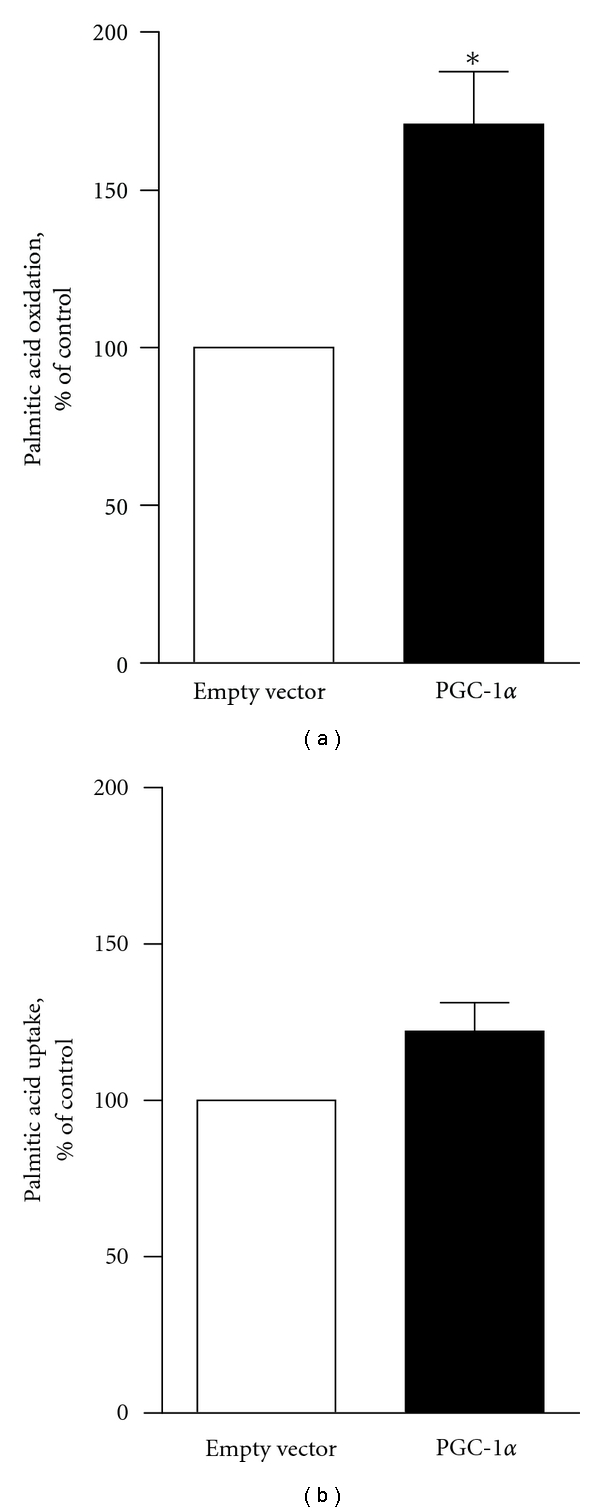
Effects of PGC-1*α* overexpression on palmitic acid oxidation (a) and uptake (b) in human myotubes. Myotubes infected with either empty vector or PGC-1*α* were incubated with [^3^H]palmitic acid and assayed for labeled acid soluble metabolites (ASM), as described in Section 2. (a) Values are presented as means ± SEM (*n* = 4 experiments, representing 3 different donors; with 3 replicates each). (b) Values are presented as means ± SEM (*n* = 3 experiments, representing 2 different donors; with 3 replicates each). *Significantly different from empty control vector at *P* < 0.05.

Oxidation of palmitic acid (PA) was measured 7-8 days after the onset of differentiation. Rate of PA oxidation, measured as acid soluble metabolites (ASM), was significantly increased by 71% (*P* < 0.05) in cells overexpressing PGC-1*α* compared to control cells infected with empty vector (Figure 3(a)). PA oxidation rate in uninfected cells (4.3 ± 0.7 nmol/mg/h) did not differ from the cells infected with empty control vector (4.0 ± 0.7 nmol/mg/h), showing that the observed increase in fatty acid oxidation rate (6.8 ± 1.4 nmol/mg/h) was due to overexpression of PGC-1*α*. Overexpression of PGC-1*α* did not affect the uptake of PA compared to cells infected with empty control vector (Figure 3(b)).

### 3.3. PGC-1*α* Overexpression Increased mRNA Expression of Genes Important for Lipid Metabolism, Mitochondrial Biogenesis, and Function

**Figure 4:**
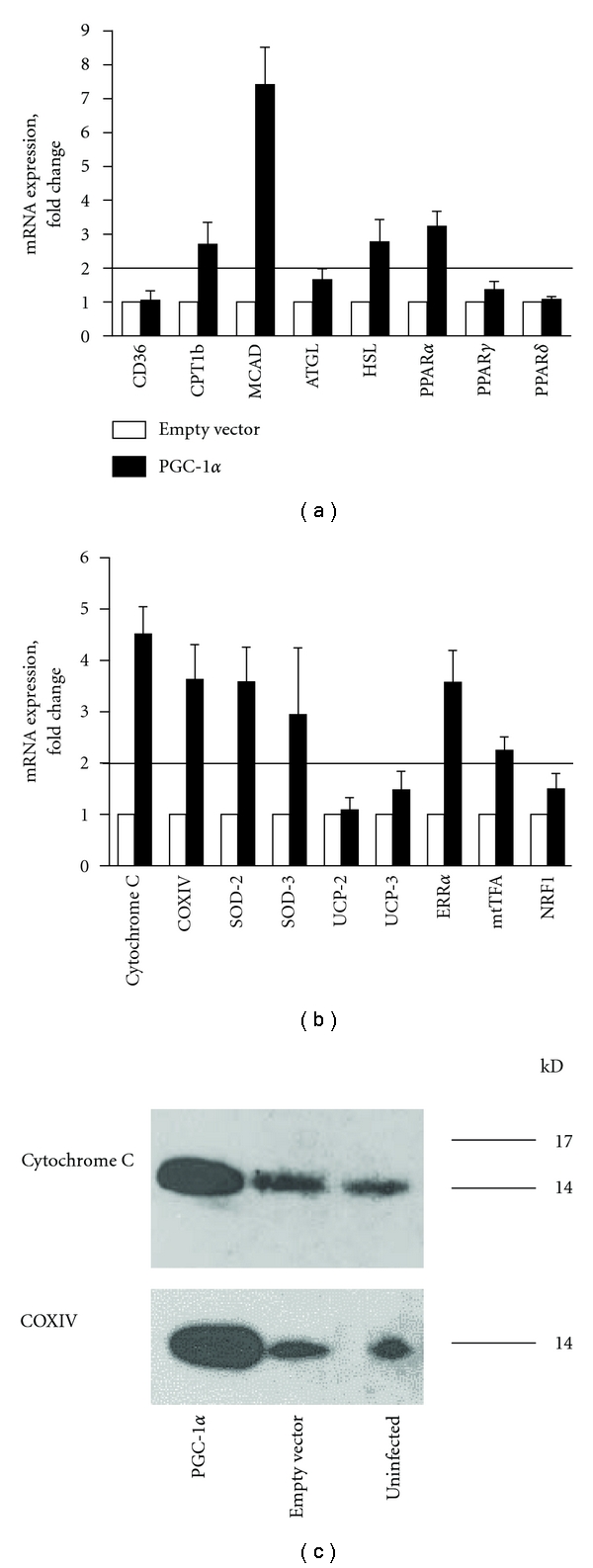
Effects of PGC-1*α* overexpression on mRNA expression of genes involved in lipid metabolism (a), mitochondrial function and biogenesis (b) and on protein levels of mitochondrial components cytochrome C and COXIV (c). (a) and (b): mRNA was isolated from cultured myotubes infected with either empty control or PGC-1*α* eight days after the onset of the differentiation. Expression was assessed by RT-PCR as described in Section 2, and values are presented as means ± SEM of 3 experiments, each representing one donor, with 3 replicates each, normalized to levels of the housekeeping gene 36B4. A fold change ≥2 or ≤0.5 was considered an increase or decrease in expression level, respectively. (c): Aliquotes from total cell lysates were electrophoretically separated and immunoblotted with specific antibodies as described in Section 2. Images represent a single experiment.

To identify potential mechanisms responsible for the observed effects of PGC-1*α* overexpression on PA oxidation, mRNA expressions of selected genes encoding key enzymes regulating fatty acid transport, storage and oxidation pathways and mitochondrial function were examined (Figure 4).

Among genes involved in lipid metabolism, PGC-1*α*-mediated increase in mRNA expression was most distinct for MCAD (medium chain acyl-coenzyme A dehydrogenase) (7.4-fold), but also PPAR*α* was increased 3.2-fold, and CPT1b (carnitine palmitoyltransferase 1b) and HSL (hormone sensitive lipase) both 2.7-fold (Figure 4(a)). In comparison, mean mRNA expression levels of MCAD, PPAR*α* and CPT1b in skeletal muscle biopsies were 24-, 22.5- and 191-fold higher than in cultured cells, respectively (Table 1). Levels of PPAR*γ*, PPAR*δ*, and fatty acid transport protein CD36 were not affected by PGC-1*α* overexpression in human myotubes (Figure 4(a)). 

**Figure 5:**
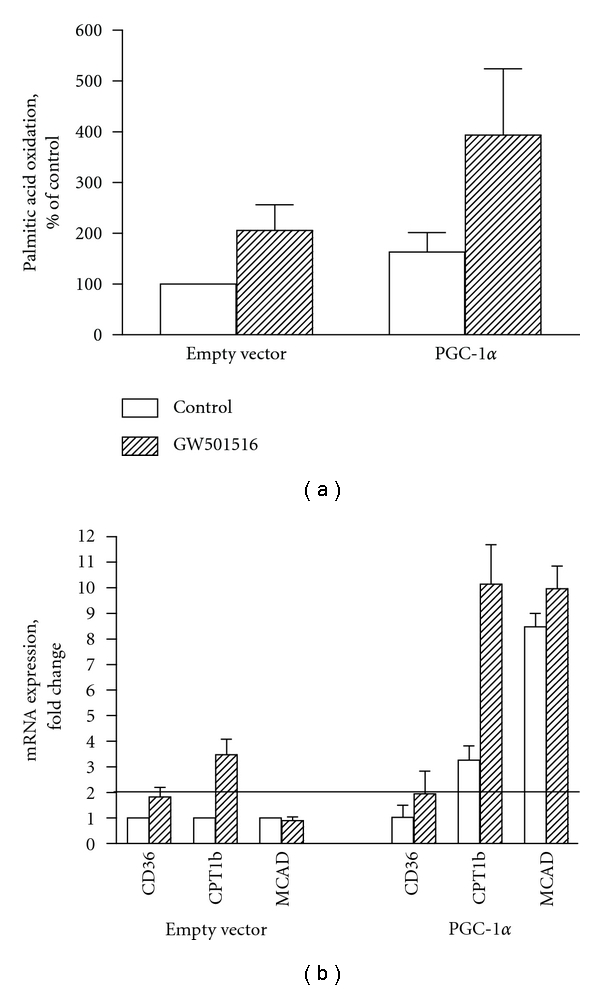
Effects of GW501516-treatment in human myotubes overexpressing PGC-1*α* on palmitic acid oxidation (a) and mRNA levels of genes involved in lipid and glucose metabolism (b). Myotubes infected with either empty vector or PGC-1*α* were treated with 10 nM GW501516 or control (0.1% DMSO) for 48 h. (a): The cells were incubated with 1 mL/well of [^3^H]palmitic acid and assayed for labeled acid soluble metabolites (ASM), as described in Section 2. Values are presented as means ± SEM (*n* = 2 experiments, each representing one donor, with 3 replicates each). (b): mRNA expression was assessed by RT-PCR as described in Section 2. Values are presented as means ± SEM (*n* = 2 experiments, each representing one donor, with 3 replicates each), normalized to the levels of housekeeping gene 36B4 in control cells infected with empty vector and treated with DMSO. A fold change ≥2 or ≤0.5 was considered an increase or decrease in expression level, respectively.

Overexpression of PGC-1*α* resulted in a marked increase in mRNA levels of several important components of the respiratory chain: cytochrome C (4.5-fold), COX IV (cytochrome C oxidase IV) (3.6-fold), SOD-2 (superoxide dismutase-2) (3.6-fold), and SOD-3 (2.9-fold) (Figure 4(b)). Protein levels of cytochrome C and COXIV were also increased in cells overexpressing PGC-1*α* compared to both cells infected with empty control vector and uninfected cells (Figure 4(c)). Two important genes involved in regulation of mitochondrial biogenesis, ERR*α* (estrogen related receptor *α*) and mtTFA (mitochondrial transcription factor A), were also noticeably increased in PGC-1*α* cells, by 3.6-fold and 2.2-fold, respectively (Figure 4(b)). Expressions of NRF1 (nuclear respiratory factor 1) and uncoupling proteins UCP-2 and UCP-3 did not appear to be affected by PGC-1*α* overexpression (Figure 5(b)). In biopsies, mean mRNA expression levels of cytochrome C, COX IV, and ERR*α* were 9.1-, 5.0-, and 7.0-fold higher than in cultured cells, respectively (Table 1).

### 3.4. PPAR*δ* Stimulation Potentiated PGC-1*α*-Induced Palmitic Acid Oxidation

We further examined whether effects of PGC-1*α* overexpression on fatty acid metabolism in human myotubes could be potentiated by simultaneously stimulating PPAR*δ*, a known target of PGC-1*α* coactivation [25]. We used a highly potent and selective PPAR*δ* agonist, GW501516 [40], which we have previously shown to increase the rate of oleic acid oxidation in cultured human skeletal muscle cell [41]. In myotubes overexpressing PGC-1*α*, 48 h of GW501516 (10 nM) treatment seemed to potentiate PA oxidation (from 7.6 ± 1.7 nmol/mg/h to 18.2 ± 5.9 nmol/mg/h) compared to myotubes infected with empty vector (from 4.6 ± 0.1 nmol/mg/h to 9.5 ± 2.2 nmol/mg/h) (Figure 5(a)). At the mRNA level, there was an additive effect of PPAR*δ* activation by GW501516 on PGC-1*α*-induced CPT1b expression (Figure 5(b)). Expression of genes involved in mitochondrial function (cytochrome C, COXIV, ERR*α*, UCP-2, and UCP-3) was not affected by GW501516 treatment (data not shown).

### 3.5. Overexpression of PGC-1*α* Decreased Glucose Transport

**Figure 6:**
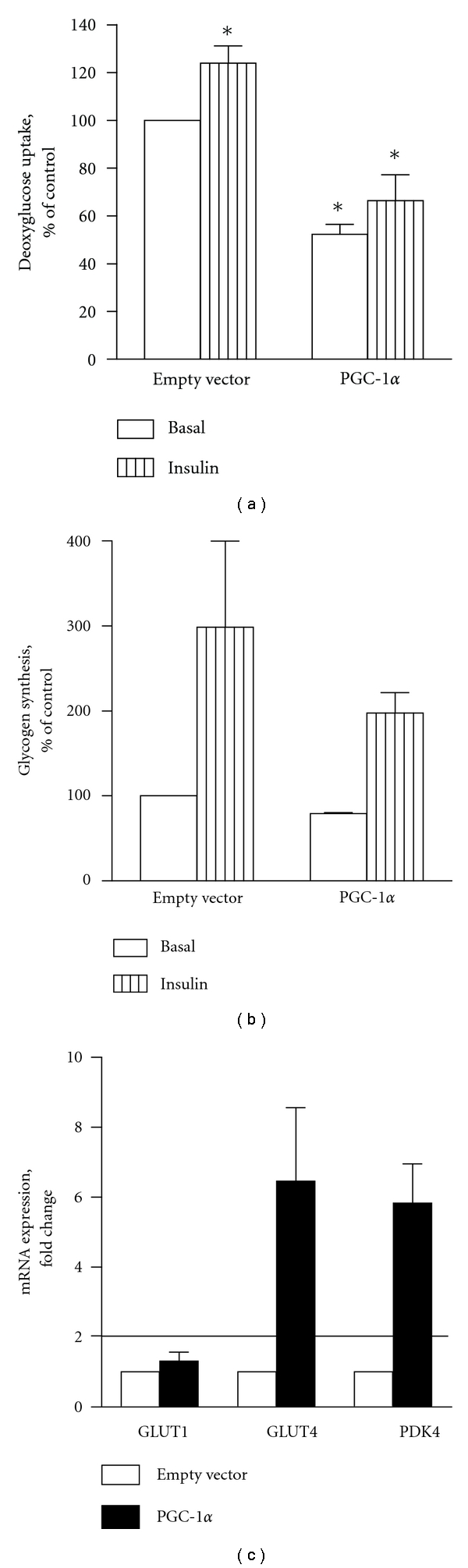
Deoxyglucose transport (a), glycogen synthesis (b) and mRNA expression (c) of GLUT1, GLUT4 and PDK4 in human myotubes infected with either empty vector or PGC-1*α*. (a): Cultured myotubes infected with either empty vector or PGC-1*α* were incubated with 1 mL/well of serum-free DMEM ± cytochalasin B 10 *μ*M for 60 min, followed by a 15 min exposure to 2-deoxy-D-[^3^H]glucose (1 *μ*Ci/mL) with or without cytochalasin B, and with or without insulin, as described in Section 2. Values are normalized to the levels of uptake in control cells infected with empty vector and presented as means ± SEM of 4 experiments, representing 3 different donors, with 3 replicates each. *Statistically significant compared to basal uptake in control cells infected with empty vector (*P* < 0.05) (b): Myotubes infected with either empty vector or PGC-1*α* were incubated for 2 h in serum-free DMEM (± insulin), and then exposed to D-[^14^C(U)]glucose ± insulin. After 60 min, the cells were washed three times with ice-cold PBS and lysed with 1 M NaOH. Synthesised glycogen was measured as described in Section 2. Values are presented as means ± SEM of 2 experiments, each representing one donor, with 3 replicates each. (c): mRNA expression of GLUT1, GLUT4 and PDK4. mRNA was isolated from cultured myotubes infected with either empty control vector or PGC-1*α* eight days after the onset of differentiation. Expression was assessed by RT-PCR, and values are presented as means ± SEM of 3 experiments, each representing one donor, with 3 replicates each, normalized to the levels of housekeeping gene 36B4. A fold change ≥2 or ≤0.5 was considered an increase or decrease in expression level, respectively.

To further assess metabolic effects of PGC-1*α* overexpression, glucose transport and glycogen synthesis were measured. In cells overexpressing PGC-1*α*, deoxyglucose uptake was decreased compared to cells infected with empty vector (Figure 6(a)). The rate of deoxyglucose transport was unchanged in cells infected with empty control vector compared to uninfected cells (31.7 ± 8.9 nmol/mg/min and 33.3 ± 9.3 nmol/mg/min, resp.), while it decreased to 15.6 ± 8.9 nmol/mg/min in myotubes overexpressing PGC-1*α*. Insulin increased glucose uptake by about 25% both in control cells and in cells overexpressing PGC-1*α* (Figure 6(a)). Glycogen synthesis was also induced by insulin, but neither basal nor insulin-induced glycogen synthesis were affected by PGC-1*α* overexpression (Figure 6(b)). The level of GLUT1 mRNA was unaffected by PGC-1*α* expression, while GLUT4 mRNA level increased 6-fold (Figure 6(c)). PDK4 (pyruvate dehydrogenase kinase 4) was also markedly increased in PGC-1*α* infected cells (6-fold) (Figure 6(c)).

### 3.6. PGC-1*α* Overexpression Reduced Expression of Fiber Type IIa Gene Marker

**Figure 7:**
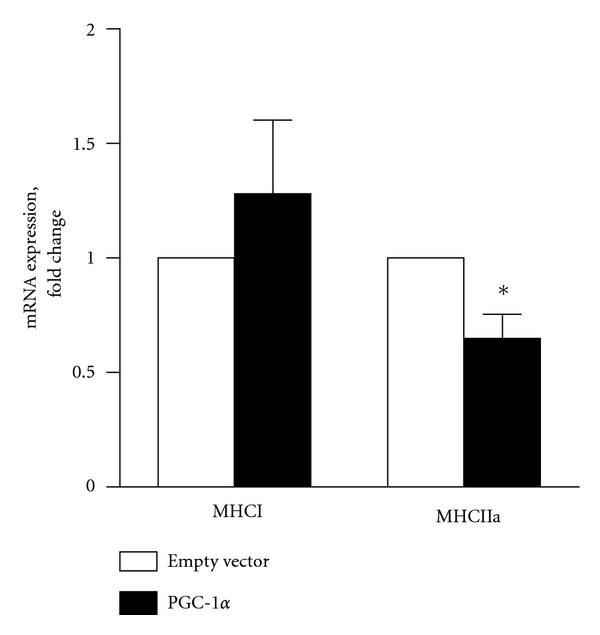
Effects of PGC-1*α* overexpression on mRNA expression of fiber-type marker genes MHCI and MHCIIa. mRNA was isolated from cultured myotubes infected with either empty control vector or PGC-1*α*, eight days after the onset of differentiation. Gene expression was assessed by RT-PCR, and values are presented as means ± SEM of 3 experiments, each representing one donor, with 3 replicates each, normalized to the levels of housekeeping gene 36B4. *Significantly different from cells infected with empty control vector at *P* < 0.05.

To assess the role of PGC-1*α* on fiber-type gene markers in myotubes, we further investigated expression of genes specifically enriched in either type I (slow) fibers (MHCI) or type IIa (fast) fibers (MHCIIa). Expression of MHCIIa mRNA was significantly decreased in cells overexpressing PGC-1*α* (*P* < 0.005) (Figure 7), while the increase in MHCI mRNA level was not significant. Thus, the MHCI/MHCIIa mRNA ratio was nearly doubled in cells overexpressing PGC-1*α* compared to control cells infected with empty vector (from 3.4 to 5.9, resp.).

## 4. Discussion

The aim of the present study was to determine whether overexpression of the transcriptional coactivator PGC-1*α* would increase oxidative capacity of human skeletal muscle cells and to compare these cells to skeletal muscle *in vivo*. PGC-1*α* overexpression increased palmitic acid oxidation and expression of key genes involved in regulation of lipid metabolism: CPT1b, MCAD, PPAR*α* and HSL, as well as genes involved in mitochondrial function and biogenesis. Compared to skeletal muscle *in vivo*, PGC-1*α* overexpression increased expression of several genes which were downregulated during the process of cell isolation and culturing. Further, our results showed that uptake of glucose in skeletal muscle cells infected with PGC-1*α* was decreased, while mRNA expression of PDK4 was increased, as well as mRNA level of GLUT4. Moreover, when PGC-1*α* was overexpressed, the ratio of the mRNA level of MHCI (a gene marker of type I, slow oxidative fiber type) to that of MHCIIa (a gene marker of glycolytic, fast-twitch skeletal muscle fibers) was increased.

Human primary myotubes retain some of the metabolic characteristics of mature skeletal muscles, but are generally limited, *in vitro*, by their low oxidative capacity [29, 30]. This is possibly due to downregulation of PGC-1*α* and lack of proliferation of mitochondria in the absence of necessary cell environmental signals. In the present study, the rate of palmitic acid oxidation was significantly increased when PGC-1*α* was overexpressed; an effect which cannot be attributed to increased uptake of palmitic acid from the medium, as this was unchanged. The increase in the oxidation rate of palmitic acid was accompanied by enhanced expression of key genes regulating lipid oxidation: CPT1b, MCAD, and PPAR*α*. ATGL has been shown to be upregulated in human skeletal muscle after exercise [42], along with increased utilization of intramyocellular triacylglycerols, while protein levels of HSL remain unchanged [42, 43]. In the present work, ATGL and HSL mRNA levels were increased by 1.6 and 2.4 fold, respectively, when PGC-1*α* was overexpressed. Interestingly, an *in vivo *study has shown that in human skeletal muscles, ATGL is expressed exclusively in type I (oxidative) fibers [44], so upregulation of this gene could indicate a shift toward more fiber type I-like myotubes when PGC-1*α* is overexpressed.

PGC-1*α* has been shown to increase mitochondrial biogenesis by several cellular mechanisms [10, 26, 45–47] and to improve lipid oxidation at rest and during submaximal exercise. We found several components of the respiratory chain to be increased at the mRNA level, such as cytochrome C and COXIV, and increase in protein levels of these two components were also compared by immunoblotting (Figure 4(c)). mtTFA, a transcription factor downstream of PGC-1*α*, critical for mtDNA replication [48], was also increased, suggesting that biogenesis of mitochondria might be increased when PGC-1*α* is overexpressed in cultured human myotubes. Furthermore, both superoxide dismutases (SODs) investigated in the present study, the mitochondrial SOD-2 and the extracellular SOD-3, were upregulated at mRNA level when PGC-1*α* was overexpressed. SODs catalyze the reduction of superoxide anions into hydrogen peroxide and oxygen, contributing as such to antioxidant defense of nearly all cells exposed to oxygen, and recent findings have shown that SOD-2 overexpression in mice preserves myoblast mitochondrial mass and function with aging [49]. 

Recently, PGC-1*α* and PPAR*δ* have been identified as key players in exercise-signaling cascade leading to mitochondrial biogenesis [16, 27]. In the present work, we also wanted to investigate whether some of the effects of PGC-1*α* overexpression on lipid metabolism and mitochondrial function could be potentiated by simultaneously activating endogenous PPAR*δ* with a selective agonist GW501516. PPAR*δ* stimulation appeared to enhance PGC-1*α*-mediated increase in palmitic acid oxidation and CPT1b expression. The effect of PPAR*δ* activation on expression of CPT1b is in agreement with results from *in vitro* studies using mouse myotubes [50]. In our study, we could not see any effect of PPAR*δ* activation on the expression of genes in mitochondrial function, which is in contrast to what has been reported in the muscle-specific VP16-PPAR*δ* transgenic mice but not in the transgenic mice overexpressing the native PPAR*δ* protein [27], and in agreement with results found in C2C12 cells (skeletal mouse muscle cells) [50], PPAR*δ* activation did not promote mitochondrial gene expression, even though it increased fatty acid oxidation, since none of the genes in electron transport chain were increased by PPAR*δ* stimulation. 

Both PGC-1*α* and GLUT4 are often deficient in cultured skeletal muscle cells [24, 34], and in primary human myotubes, basal glucose uptake is generally mediated by other glucose transporters, such as GLUT1 and GLUT3 [51, 52]. In the present work, basal glucose uptake was significantly decreased in human myotubes overexpressing PGC-1*α*, while GLUT1 mRNA expression was unchanged. A previous study with adenoviral overexpression of PGC-1*α* in C2C12 and L6 cells showed total restoration of GLUT4 mRNA levels to those observed *in vivo*, with a 3-fold increase in insulin-stimulated glucose transport [24]. In our study, overexpression of PGC-1*α* caused increased GLUT4 mRNA but did not increase insulin-stimulated glucose uptake. Inconsistencies between mRNA levels of GLUTs and functional data have been reported in human cultured skeletal muscle cells previously [53–55]. However, it is possible that the observed decrease in glucose uptake could be due to increased mRNA level of PDK4, an inhibitor of pyruvate dehydrogenase complex, which switches oxidation towards lipids.

Muscle fibre type and oxidative capacity have been linked to obesity and insulin resistance with a higher percentage of type 1 being positively related to insulin action and inversely with obesity [56, 57]. In addition it has been shown in studies with transgenic animals that PGC-1*α* appears to be an important factor in regulating muscle fiber type determination [16]. In the present study, myotubes infected with retro virus coding for PGC-1*α* had in increased MHCI/MCHIIa ratio based on gene markers for type I and type IIa fibers respectively. To our knowledge, shift in fiber-type composition has not previously been shown in cultured human skeletal muscle cells. The fact that there is a tendency towards increased MHCI/MHCIIa ratio indicates the possibility of a more complete switching between fiber types at conditions when PGC-1*α* is overexpressed, also in cultured skeletal muscle cells. 

In summary, in the present study, overexpression of PGC-1*α* in cultured human myotubes increased fatty acid oxidative capacity of the cells and increased expression of genes involved in regulation of mitochondrial function. Compared to skeletal muscle *in vivo*, PGC-1*α* overexpression increased expression of several genes, which were downregulated during the process of cell isolation and culturing. We have also shown that mRNA expression of a fast fiber-type gene marker (MHCIIa) was decreased, suggesting that PGC-1*α* may play a role in fiber-type regulation in human myotubes.

## Figures and Tables

**Table 1:** Ratio of mean mRNA expression levels in skeletal muscle biopsies compared to cell cultures. mRNA was isolated from muscle biopsies and cultured myotubes, and expression was assessed by RT-PCR, as described in Materials and Methods. Values are presented as mean ± SEM of 4 experiments, each representing one donor, with 3 replicates each, and normalized to levels of the housekeeping gene 36B4.

Gene	Ratio biopsy/cells (+/− SEM)
COXIV	5.0 (0.34)
CPT1b	191.1 (52.7)
Cytochrome C	9.1 (0.38)
ERR*α*	7.0 (0.5)
MCAD	24.0 (1.7)
PDK4	1203 (300)
PPAR*α*	22.5 (7.3)
PPAR*δ*	1.6 (0.23)
PPAR*γ*	6.5 (2.7)
