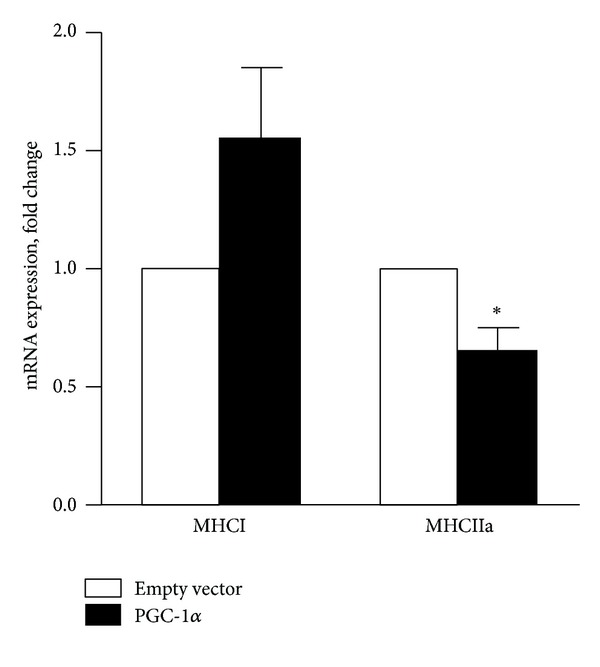# Erratum to “Overexpression of PGC-1***α*** Increases Fatty Acid Oxidative Capacity of Human Skeletal Muscle Cells”

**DOI:** 10.1155/2013/347567

**Published:** 2013-03-26

**Authors:** Nataša Nikolić, Magdalena Rhedin, Arild C. Rustan, Len Storlien, G. Hege Thoresen, Maria Strömstedt

**Affiliations:** ^1^Department of Pharmaceutical Biosciences, School of Pharmacy, University of Oslo, P.O. Box 1068 Blindern, N-0316 Oslo, Norway; ^2^AstraZeneca Reseach and Development, SE-43185 Mölndal, Sweden; ^3^Boden Institute of Obesity, Nutrition and Exercise, University of Sydney, Sydney, NSW 2006, Australia

Unfortunately there was a mistake in Figure 7. The primer used for mRNA expression was actually MYH1, which regulates expression of MHC type IIx muscle fibers and not MHC type I as stated in the figure. However, we have repeated the experiment with the correct primer MYH7 (acc_no. NM000257.2, F: CTCTGCACAGGGAAAATCTGAA, R: CCCCTGGAGACTTTGTCTCATT), and the new Figure 7 is shown here. This makes no difference to the conclusions of the paper. There was no significant change in mRNA of MHCI (MYH7), neither was the MHCI/MHCIIa mRNA ratio significantly increased. However, the last sentence in Section 3 (page 7) should be slightly changed: “Thus, the MHCI/MHCIIa mRNA ratio was approximately doubled in cells overexpressing PGC-1*α*  compared to control cells infected with empty vector (from 4.5 to 9.2, resp.).”

## Figures and Tables

**Figure 7:**